# NEIGHBORHOOD SOCIOECONOMIC STATUS AND GRAY MATTER VOLUME IN OLDER ADULTS

**DOI:** 10.1093/geroni/igz038.1542

**Published:** 2019-11-08

**Authors:** Sara L Godina, Caterina Rosano, Peter J Gianaros, Howard J Aizenstein, Michelle C Carlson, Philippa Clarke, Andrea L Rosso

**Affiliations:** 1 University of Pittsburgh, Pittsburgh, Pennsylvania, United States; 2 Johns Hopkins University, Baltimore, Maryland, United States; 3 University of Michigan, Ann Arbor, Michigan, United States

## Abstract

Lower neighborhood socioeconomic status (nSES) is associated with poorer cognitive function; underlying neural correlates are unknown. Cross-sectional associations of nSES (six census-derived measures of income, education, and occupation) and gray matter volume (GMV) of eight memory-related regions (hippocampus, middle frontal gyrus, amygdala, insula, parahippocampal gyrus, anterior, middle, and posterior cingulum) were examined in 264 community-dwelling older adults (mean age=83, 56.82% female, 39.02% black). In linear mixed effects models adjusted for total brain atrophy and accounting for geographic clustering, higher nSES was associated with greater GMV of the left hippocampus, left posterior cingulum, and bilateral insula, middle frontal, and parahippocampal gyri. nSES remained associated with GMV of the right insula (β= -32.26, p=0.026, 95%CI: -60.66, -3.86) after adjusting for individual level age, gender, race, income, and education. The nSES and cognitive function association may not be due to gray matter volume differences; other behavioral and biological mediators should be explored.

